# Receptor-Enriched Analysis of functional connectivity by targets (REACT): A novel, multimodal analytical approach informed by PET to study the pharmacodynamic response of the brain under MDMA

**DOI:** 10.1016/j.neuroimage.2019.04.007

**Published:** 2019-07-15

**Authors:** Ottavia Dipasquale, Pierluigi Selvaggi, Mattia Veronese, Anthony S. Gabay, Federico Turkheimer, Mitul A. Mehta

**Affiliations:** Department of Neuroimaging, Institute of Psychiatry, Psychology & Neuroscience, King's College London, London, United Kingdom

**Keywords:** MDMA, Resting state fMRI, Functional connectivity, Pharmacological neuroimaging, Serotonin, Pharmacodynamic response

## Abstract

One of the main limitations of pharmacological fMRI is its inability to provide a molecular insight into the main effect of compounds, leaving an open question about the relationship between drug effects and haemodynamic response. The aim of this study is to investigate the acute effects of 3,4-methylenedioxymethamphetamine (MDMA) on functional connectivity (FC) using a novel multimodal method (Receptor-Enriched Analysis of functional Connectivity by Targets - REACT). This approach enriches the resting state (rs-)fMRI analysis with the molecular information about the distribution density of serotonin receptors in the brain, given the serotonergic action of MDMA.

Twenty healthy subjects participated in this double-blind, placebo-controlled, crossover study. A high-resolution in vivo atlas of four serotonin receptors (5-HT_1A_, 5-HT_1B_, 5-HT_2A_, and 5-HT_4_) and its transporter (5-HTT) was used as a template in a two-step multivariate regression analysis to estimate the spatial maps reflecting the whole-brain connectivity behaviour related to each target under placebo and MDMA.

Results showed that the networks exhibiting significant changes after MDMA administration are the ones informed by the 5-HTT and 5-HT_1A_ distribution density maps, which are the main targets of this compound. Changes in the 5-HT_1A_-enriched functional maps were also associated with the pharmacokinetic levels of MDMA and MDMA-induced FC changes in the 5-HT_2A_-enriched maps correlated with the spiritual experience subscale of the Altered States of Consciousness Questionnaire.

By enriching the rs-fMRI analysis with molecular data of voxel-wise distribution of the serotonin receptors across the brain, we showed that MDMA effects on FC can be understood through the distribution of its main targets. This result supports the ability of this method to characterise the specificity of the functional response of the brain to MDMA binding to serotonergic receptors, paving the way to the definition of a new fingerprint in the characterization of new compounds and potentially to a further understanding to the response to treatment.

## Highlights

• MDMA connectivity effects understood through the distribution of 5-HT_1A_ and 5-HTT.
• Direct link between PK levels of MDMA and 5-HT_1A_-enriched functional connectivity maps.
• Ability to link receptor targets to functional mechanisms underlying behaviour.
• Mapping pharmacodynamic effects onto the drug's molecular targets.

## Graphical abstract

**Figure:**
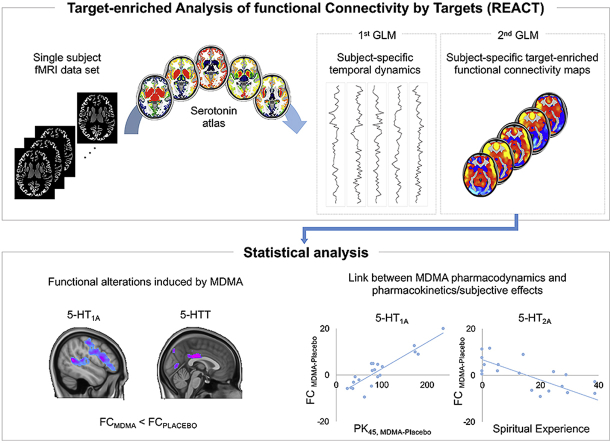

## 1 Introduction

3,4-methylenedioxymethamphetamine (MDMA) increases levels of serotonin, dopamine and noradrenaline in the brain (Liechti and Vollenweider, 2001). This drug has a history of recreational use because of its positive effects on mood and social interaction, which include feelings of empathy and warmth for others, sociability and a sense of being at peace with the world, as well as euphoria (Rodgers et al., 2006; Sumnall et al., 2006). It can also produce mild visual hallucinatory phenomena, which have been linked to the 5-HT_2A_ receptor, the same receptor targeted by the classic psychedelics (Gouzoulis-Mayfrank et al., 1996; Harris et al., 2002; Liechti and Vollenweider, 2001). While currently classed in the list of Schedule I drugs, indicating no potential medicinal benefits, preliminary evidence of a potential clinical use of MDMA for mental illness (Amoroso and Workman, 2016; Mithoefer et al., 2013) has recently re-ignited interest in its pharmacology, behavioural effects and mechanisms of action.

The serotonergic pharmacology of MDMA is primarily characterised by the inhibition of 5-HT reuptake (Iravani et al., 2000) and the stimulation of 5-HT release in the extracellular space via the serotonin transporter (5-HTT) (Bradbury et al., 2014; Gudelsky and Nash, 1996; Nichols, 1986). Other studies have also shown a weaker affinity of MDMA to 5-HT_1A_ and 5-HT_2A_ receptor sites (Green et al., 2003; Ray, 2010) as well as non-serotonergic effects (Hasler et al., 2009). Some behavioural effects of MDMA such as feelings of empathy, sociability, and interpersonal closeness (Rodgers et al., 2006; Sumnall et al., 2006) are thought to be induced in part by an increase in the levels of the peptide oxytocin (Dumont et al., 2009; Hysek et al., 2012) with 5-HT_1A_ receptor playing an important role in this mechanism (Mottolese et al., 2014; Thompson et al., 2007). .

While MDMA pharmacology has been extensively investigated in terms of its primary targets and affinities, its effect on brain function is less well understood. The investigation of MDMA-induced effects on brain function using resting state fMRI (rs-fMRI) has been focused on specific regions related to social and affective processing, namely the insula, ventromedial prefrontal cortex, hippocampus and amygdala (Carhart-Harris et al., 2015; Walpola et al., 2017). These studies revealed changes in functional connectivity (FC) for circuitry implicated in anxiety and stress-related disorders such as the medial prefrontal cortex, medial temporal regions, amygdala and hippocampus (Carhart-Harris et al., 2015) and a reduction in insula connectivity which correlated with the acute subjective experiences of altered bodily sensations and baseline trait anxiety (Walpola et al., 2017). An independent component analysis (ICA) was performed on the same dataset (Carhart-Harris et al., 2015; Walpola et al., 2017) to evaluate the effects of MDMA on FC between the resting state networks (RSNs). This analysis revealed an increased FC between the executive control network the anterior default mode network (Roseman et al., 2014).

One of the main limitations of pharmacological fMRI is its inability to provide a molecular insight into the main effect of compounds. In fact, this imaging technique relies on the strong assumption that haemodynamic changes can be considered a proxy of altered neurotransmission due to pharmacological agonism or antagonism actions (Attwell and Iadecola, 2002), but the fMRI signal has no intrinsic selectivity to any particular receptor sites. Therefore, the degree to which the fMRI response indexes the action at drug target sites, which could be useful in parsing mechanistic underpinnings, is still an open question.

Here we propose Receptor-Enriched Analysis of functional Connectivity by Targets (REACT), a method that utilises the molecular information about target distribution provided by Positron Emission Tomography (PET) to enrich the pharmacological rs-fMRI analysis. In particular, we tested the degree to which the haemodynamic response to MDMA mirrors the 5-HT receptor density profiles, given its known mixed profile of serotonergic action. We used a publicly available atlas (Beliveau et al., 2017), which provides the 5-HT receptor density profiles for 5-HT_1A_, 5-HT_1B_, 5-HT_2A_ and 5-HT_4_ receptors and the 5-HTT transporter, as target-site distribution maps in a two-step multivariate regression analysis and estimated the spatial maps reflecting the whole-brain connectivity behaviour related to each target, in order to link the knowledge about their distribution with the main effect of MDMA on FC. All the available PET maps were included in the model as we wanted to test the ability of this method to characterise the specificity of the functional response of the brain to MDMA binding to serotonergic receptors. Given MDMA affinity profiles and proposed mediators of MDMA effects at the 5-HTT transporter, the 5-HT_1A_ and 5-HT_2A_ receptor targets (Bradbury et al., 2014; Green et al., 2003; Gudelsky and Nash, 1996; Liechti and Vollenweider, 2001; Nichols, 1986; Ray, 2010; Thompson et al., 2007) we hypothesised that FC informed by these three target maps will be sensitive to MDMA administration.

In addition to the derivation of the spatial maps according to each receptor density distribution, it is possible that the interaction between the targets has a significant influence on FC. For example, both the 5-HTT and the 5-HT_1A_ are important for serotoninergic regulation and their in vivo receptor densities are related both within the brain stem (e.g. raphe nucleus) and at the more distal sites (Bose et al., 2011). Therefore, we set out to explore whether or not overlapping expressions of serotonergic targets are also associated with the main effect of MDMA on FC.

Finally, we investigated whether subjective effects and pharmacokinetic (PK) measures, such as MDMA plasma levels and MDMA-induced increases in the plasma levels of oxytocin, are linked with the pharmacodynamic effects of MDMA in the brain, exploring possible relationships with the 5-HT receptor distribution.

## 2 Methods

### 2.1 Participants

Twenty-one male participants were recruited from the community and passed screening, which included prior experience of MDMA/ecstasy. Twenty of these participants completed the study (mean age 24.8 y, SD = 3.7, range = 21–37), while one participant withdrew from it after his first visit. This was unrelated to his participation and unblinding revealed that he received placebo on that visit. Participants were excluded if they had personal history of psychiatric illness, assessed with the Mini-International Neuropsychiatric Interview (Sheehan et al., 1998); first-order relative with a history of psychotic illness; evidence of cardiac (assessed with ECG), hepatic, renal, gastrointestinal (assessed with standard blood screening) or neurological disorders; excessive use of caffeine (>six cups of coffee per day) and alcohol (>28 units per week); current use of medication; failure of drugs of abuse test at screening or on either study day (drugs tested for: amphetamine, barbiturates, benzodiazepines, cocaine, THC, methadone, methamphetamine, opiate, MDMA, tricyclic antidepressants). Participants were only included in this study if they had at least one previous experience with MDMA. They were also required to have not used MDMA in the three months leading up to their involvement in the study.

All participants gave written informed consent to take part in the study and were financially compensated for their time. The study received ethical approval from King's College London's Psychiatry, Nursing and Midwifery Research Ethics Committee (PNM/14/15–32). All experiments were performed in accordance with relevant guidelines and regulations.

### 2.2 Study design and behavioural analyses

Data was collected using a within-subjects, double-blind randomized, placebo-controlled, crossover design. Following a successful screening, participants attended two experimental study days at least one week apart (mean 9.3 days, SD 5.7, range 7–31). For a full description of the experimental day, see (Gabay et al., 2018).

Subjective effects were assessed using the 11-dimension Altered States of Consciousness Questionnaire (ASC), the gold standard when investigating compounds with psychedelic-like properties (Studerus et al., 2011).

MDMA plasma levels were measured 45 min post-dose and 165 min post-dose. Oxytocin plasma levels were measured at these same timepoints, as well as 15min pre-dose to obtain a baseline measure.

### 2.3 Image acquisition

MR imaging was performed on a MR750 3 T General Electric MR scanner using a 32-channel head coil. A 3D T1-weighted anatomical scan was obtained for each participant in one session using an MPRAGE acquisition (TR = 7.312 ms, TE = 3.02 ms, flip angle = 11⁰, slice thickness = 1.2 mm, 196 sagittal slices, FOV = 270 mm). Functional MRI data were obtained during rest in both sessions using a multi-echo EPI sequence (TR = 2500 ms, TEs = 12, 28, 44 ms, resolution = 3.75 × 3.75 × 4.2 mm, slice thickness = 3 mm, 27 axial slices aligned to the AC-PC line, 192 vol, flip angle = 80°, field of view: 240 mm). Participants were instructed to remain awake with their eyes open and fixate on a cross for the duration of the resting state scan. The resting state scan commenced approximately 90 min post-dose (mean 99.5 min, SD 5.5 min).

### 2.4 Image pre-processing

The rs-fMRI dataset was pre-processed using AFNI (Cox, 1996) and FMRIB Software Library (FSL). Pre-processing steps included volume re-alignment, time-series de-spiking and slice time correction. After the pre-processing, functional data were optimally combined (OC) by taking a weighted summation of the three echoes using an exponential T2* weighting approach (Posse et al., 1999). The OC data were then de-noised with the Multi-Echo ICA (ME-ICA) approach implemented by the tool meica. py (Version v2.5) (Kundu et al., 2013, 2014) to remove motion artefacts and other non-BOLD sources of noise. This de-noising method has proved its effectiveness in reducing the non-BOLD sources of noise compared to the standard regression approaches, and increasing the temporal SNR (Dipasquale et al., 2017; Kundu et al., 2013). Briefly, multi-echo principal component analysis was first used to reduce the data dimensionality in the OC dataset. Spatial ICA was then applied, and the independent component time-series were fit to the pre-processed time-series from each of the three echoes to generate ICA weights for each echo. These weights were then fitted to the linear TE-dependence and TE-independence models to generate F-statistics and component-level κ and ρ values, which respectively indicate BOLD and non-BOLD weightings. The κ and ρ metrics were then used to identify non-BOLD-like components to be regressed out of the OC dataset as noise regressors. Further technical details on ME-ICA can be found in (Kundu et al., 2015).

Data were then spatially smoothed with a with an 8-mm FWHM Gaussian kernel. WM and CSF masks were obtained from the segmentation of the subjects' structural images and eroded in order to minimize the contribution of grey matter partial volume effects. After co-registering them to each individual's fMRI space, they were used to extract the mean WM and CSF signals from each participant's pre-processed dataset. Those signals were then regressed out and a high-pass temporal filter with a cut-off frequency of 0.005 Hz was applied.

A study-specific template representing the average T1-weighted anatomical image across subjects was built using the Advanced Normalization Tools (ANTs) (Avants et al., 2011). Each participant's dataset was co-registered to its corresponding structural scan, then normalized to the study-specific template before warping to standard MNI152 space. Images were finally resampled at 1 mm^3^ and 2 mm^3^ resolution.

### 2.5 Receptor-enriched analysis of functional connectivity

**Fig. 1:**
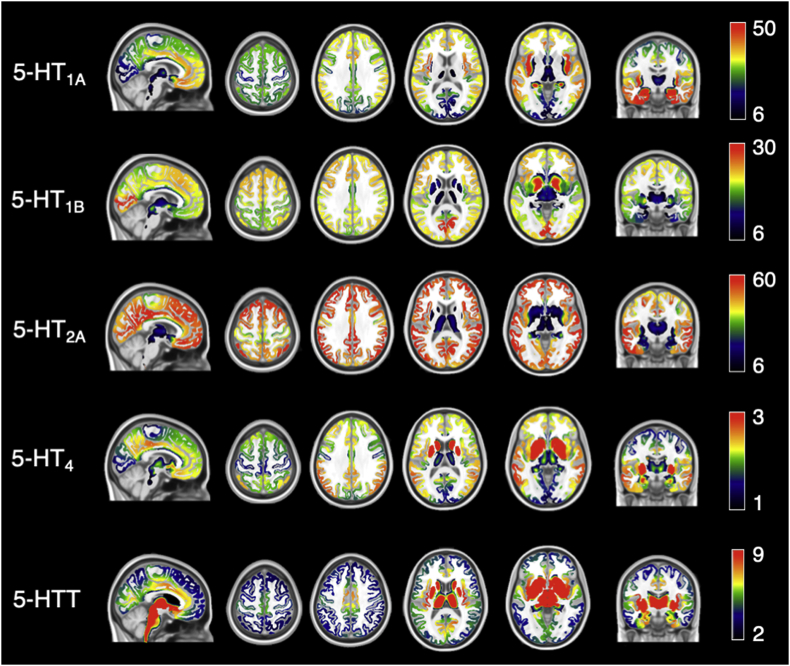
**High-resolution in vivo atlas of five serotonin targets from** (Beliveau et al., 2017). From top to bottom: 5-HT_1A_, 5-HT_1B_, 5-HT_2A_, and 5-HT_4_ receptors; 5-HTT transporter.

The high-resolution in vivo atlas (Beliveau et al., 2017) of four serotonin receptors (i.e. 5-HT_1A_, 5-HT_1B_, 5-HT_2A_, and 5-HT_4_) and the 5-HTT transporter was used to enrich the rs-fMRI analysis with the density distribution of these proteins in the healthy brain (Fig. 1). This atlas, which is freely available (https://xtra.nru.dk/FS5ht-atlas/), was created from molecular and structural high-resolution neuroimaging data consisting of PET and MRI scans acquired in 210 healthy individuals. For further details on the atlas, please refer to (Beliveau et al., 2017).

**Fig. 2:**
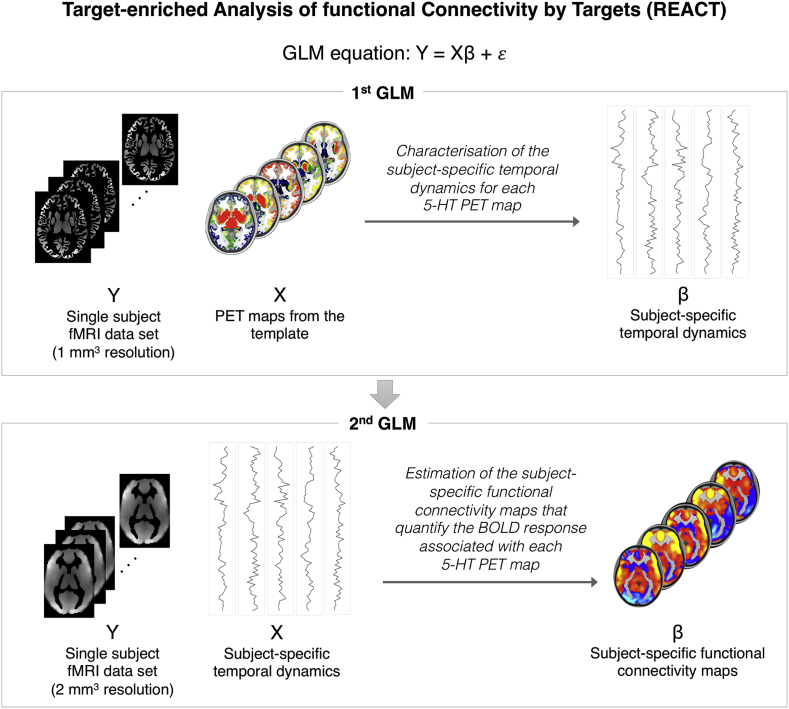
General framework of the analysis.

All available serotonin target maps entered into the first step of a two-step multivariate regression analysis (Filippini et al., 2009; Nickerson et al., 2017; Smith et al., 2014) implemented in FSL (fsl_glm command). Here, the PET maps were used as a set of spatial regressors to estimate the FC in terms of fitting the BOLD fluctuations across voxels with respect to the dominant fluctuation within these maps (Griffanti et al., 2015). As the spatial regressors and the input data need to have the same resolution, in this step the rs-fMRI images sampled at 1 × 1x1 mm^3^ were used to avoid any smoothing of the receptor maps. Both rs-fMRI data and the design matrix were demeaned (--demean option), as required when a multiple regression is performed in order to obtain a good fit. Of note, this is a standard option implemented in the dual regression approach. The rs-fMRI volumes were masked using a binarized atlas derived from the PET data to restrict the analysis to the voxels for which the receptor density information was available in the PET maps. Of note, the cerebellum was excluded from this mask as the quantification of the PET data was done using cerebellar grey matter as reference region in the kinetic model for all the radioligands (Beliveau et al., 2017). The subject-specific time series estimated in this first step were then used as temporal regressors in a second multivariate regression analysis (second step) to estimate the subject-specific spatial maps of the BOLD response after MDMA and placebo. At this stage, we used the rs-fMRI images sampled at 2 × 2x2 mm^3^ and the analysis was conducted on the whole grey matter volume. Again, both data and the design matrix were demeaned (--demean option); the design matrix columns were also normalised to unit standard deviation (--des_norm option), as usually performed in the second stage of the dual regression (Filippini et al., 2009). The general framework of this analysis is reported in Fig. 2. More technical details on the dual regression approach can be found in (Nickerson et al., 2017).

### 2.6 Interaction analysis

An additional exploratory analysis was performed to look for interactions related to co-localisation of serotonergic targets. For this analysis we computed the product of different maps and tested their non-linear combination. We focused on the targets to which MDMA has higher affinity, i.e. 5-HT_1A_, 5-HT_2A_ and 5-HTT, and performed the two-step multivariate regression analysis including in the spatial GLM (first step) these three maps and their products (i.e., 5-HT_1A_ * 5-HT_2A_, 5-HT_1A_ * 5-HTT, 5-HT_2A_ * 5-HTT and 5-HT_1A_ * 5-HT_2A_ * 5-HTT). Again, the temporal GLM (second step) included in the design matrix all the time series estimated in the first step and returned one subject-specific spatial map for every condition and PET map that entered in the model.

### 2.7 Resting state network analysis

Thirteen major RSNs were extracted from an independent rs-fMRI dataset of 43 healthy subjects (Wolke et al., 2019) collected on the same scanner (15 males, mean age 21.83 y, SD = 2.05, range = 18–25) using the group ICA implemented with MELODIC (Beckmann and Smith, 2004) with a model order of 20 (see Supplementary Fig. 2). The acquisition parameters and pre-processing pipeline correspond to those described for the dataset under exam. The components classified as RSNs were then used as a template in the dual regression (Nickerson et al., 2017) to recover the subject-specific time series and spatial maps for both conditions.

### 2.8 Statistical analysis

The subject-specific target-enriched spatial maps of the two conditions, both in the main and in the interaction analyses, and in the RSN analysis were compared using permutation tests.

Linear relationships were tested between the FC maps (MDMA minus Placebo) and subjective effects (MDMA minus Placebo), MDMA plasma levels 45 and 165 min after the dose administration (PK_45_ and PK_165_) and the MDMA and placebo difference of oxytocin peripheral levels 45 and 165 min from the administration (ΔOXT_45_ and ΔOXT_165_).

All these tests were performed with Randomise (Winkler et al., 2014), using 5000 permutations per test and contrast.

Finally, we extracted the mean FC value (MDMA minus Placebo) in the regions resulted to be significantly associated with the subjective effects, MDMA or oxytocin peripheral levels and calculated the Pearson correlation coefficients (with bootstrapping, 1000 samples) with these measures using SPSS.

## 3 Results

MDMA-induced changes in subjective effects, as measured by the ASC, and plasma oxytocin levels can be found in Gabay et al. (2019) (respectively in Fig. 2 and Table 2 of the just mentioned paper). Briefly, MDMA increased scores in 10 of the 11 dimensions of the ASC compared to placebo (spiritual experience, blissful state, insightfulness, disembodiment, impaired cognition, anxiety, complex imagery, audio/visual synaesthesia, meaning, and experience of unity; all Bonferroni-corrected *p* < 0.05). Increased oxytocin levels were seen following MDMA treatment at both timepoints (Bonferroni-corrected *p* = 0.054, 45 min post-dose; Bonferroni-corrected *p* < 0.001, 165 min post-dose). On the active treatment day, mean MDMA plasma levels were 91.7 μg/L (SD 60.2) and 188.2 μg/L (SD 32.8) at 45 min and 165 min post-dose, respectively.

### 3.1 Main analysis

**Fig. 3:**
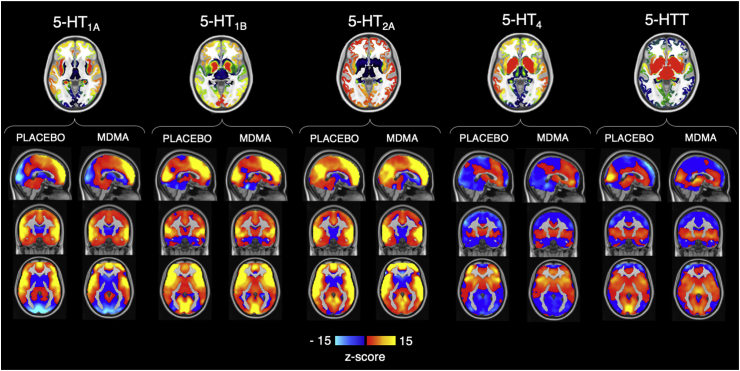
**Maps of the five serotonin targets and their respective PET-derived fMRI maps.** Top row: PET maps of the 5-HT_1A_, 5-HT_1B_, 5-HT_2A_, and 5-HT_4_ receptors and 5-HTT transporter; bottom: fMRI maps averaged across subjects for the placebo and MDMA conditions.

The two-step multivariate analysis returned one subject-specific map for each 5-HT target and drug condition. These target-enriched maps averaged across participants are reported in Fig. 3.

**Fig. 4:**
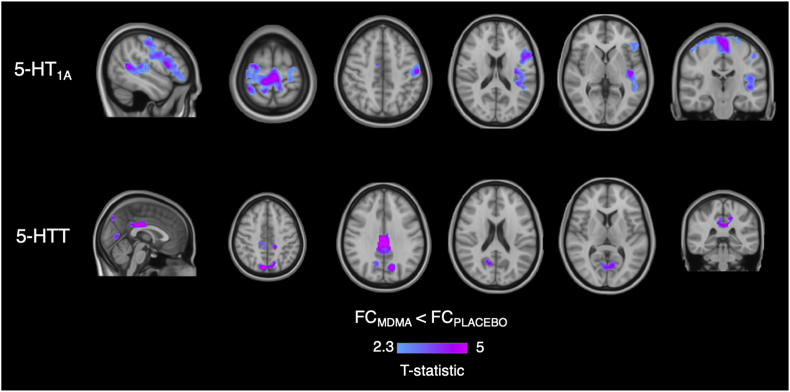
**Functional connectivity (FC) changes after MDMA in the 5-HT**_**1A**_**and 5-HTT-enriched maps.** Top row: the MDMA-induced decrease in the 5-HT1A-enriched maps is localised in the precentral and postcentral gyri, left frontal pole, left insular cortex, left inferior frontal gyrus, left middle temporal gyrus, left supramarginal gyrus, left opercular cortex, left Heschl's gyrus, left planum temporale, right superior frontal gyrus, right superior parietal lobule and right supplementary motor cortex. Bottom row: the MDMA effect reported in the 5-HTT-enriched maps is localised in the anterior and posterior cingulate gyrus, precuneous, cuneal cortex, precentral gyrus, intracalcarine and supracalcarine cortex, lingual gyrus and lateral occipital cortex. Images are shown in radiological orientation.

We found significant FC decreases (p_FWE_ < 0.05, corrected for multiple comparisons at the cluster level using the threshold-free cluster enhancement (TFCE) option, Bonferroni-corrected for multiple comparisons across maps and contrasts) induced by MDMA in the maps enriched by the 5-HT_1A_ receptor and the 5-HTT transporter distributions (Fig. 4). Specifically, the effect of MDMA on FC in the 5-HT_1A_-enriched maps involves different cortical areas including the precentral and postcentral gyri, left frontal pole, left insular cortex, left inferior frontal gyrus, left middle temporal gyrus, left supramarginal gyrus, left opercular cortex, left Heschl's gyrus, left planum temporale, right superior frontal gyrus, right superior parietal lobule and right supplementary motor cortex. The 5-HTT-enriched maps revealed an MDMA effect that was localised in the anterior and posterior cingulate gyrus, precuneus, cuneal cortex, precentral gyrus, intracalcarine and supracalcarine cortex, lingual gyrus and lateral occipital cortex. Of note, no changes in the FC maps enriched by the other targets (i.e., 5-HT_1B_, 5-HT_2A_ and 5-HT_4_) were found.

We also conducted a RSN analysis of MDMA uninformed by the 5-HT targets, the results of which are included in the supplementary materials, where we also included the results of the interaction analysis where the target-enriched maps and their products are used as regressors. Briefly, significant FC changes after MDMA compared to placebo were found in the primary visual, sensorimotor, medial temporal, salience and ventral stream networks. However, nothing survived Bonferroni correction for multiple comparisons across all the RSNs and contrasts. As regards the interaction analysis, we found a significant FC decrease induced by MDMA in the connectivity maps derived by the 5-HT_1A_ receptor, the 5-HTT transporter and the HT_1A_ – 5-HT_2A_ interaction, and an increase in the 5-HT_1A_-enriched maps (p_FWE_ < 0.05, TFCE corrected for multiple comparisons at the cluster level, Bonferroni corrected for multiple comparisons across maps and contrasts).

### 3.2 Correlation with behavioural measures, MDMA and oxytocin plasma levels

Linear relationships were tested between the FC maps derived with the REACT and subjective effects, MDMA and oxytocin plasma levels.

**Fig. 5:**
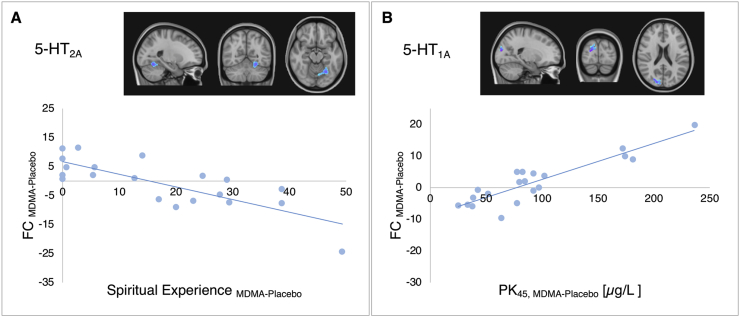
**Correlations between subjective effects/MDMA plasma levels and functional connectivity.** Panel A: an MDMA-induced FC decrease in the 5-HT_2A_-enriched maps localised in the left lingual gyrus and occipital and temporo-occipital fusiform gyrus is significantly correlated with the spiritual experience subscale. Panel B: MDMA-induced FC increases in the right supramarginal gyrus, lateral occipital cortex and cuneal cortex of the 5-HT1A-enriched maps were significantly correlated with increased MDMA plasma levels collected 45 min after the dose administration. Images are shown in radiological orientation.

MDMA-induced FC change in the 5-HT_2A_-enriched maps correlated with the spiritual experience subscale (r = - 0.782, p < 0.0001, 95% CI = −0.909 to −0.572; Fig. 5A) in the left lingual gyrus and occipital and temporo-occipital fusiform gyrus, such that those with the greatest increases in spiritual experience had the greatest reductions in connectivity for the 5-HT_2A_ target-enriched maps.

No linear relationships were found between FC maps and the oxytocin plasma levels, while MDMA-induced FC increases in some regions of the 5-HT_1A_-enriched maps were significantly correlated with increased plasma levels 45 min after MDMA administration (r = 0.9, p < 0.0001, 95% CI = 0.71 to 0.965; Fig. 5B). The correlation between FC and PK_45_ levels was localised in the right supramarginal gyrus, lateral occipital cortex and cuneal cortex.

For exploratory purposes, the same analysis was performed in the maps obtained in the interaction and RSN analyses. The results from the interaction analysis support those reported above, i.e. a correlation of the FC decrease after MDMA in the 5-HT_2A_-enriched maps with the spiritual experience subscale. Significant correlations were also found between MDMA-induced FC decreases in some regions of the 5-HT_1A_, HT_1A_ – 5-HTT and HT_1A_ – 5-HT_2A_ – 5-HTT maps and increased oxytocin plasma levels 165 min after MDMA administration. These results are detailed in the supplementary materials.

As regards the RSNs, no significant correlations with the subjective effects, MDMA and oxytocin plasma levels were found.

## 4 Discussion

To date, the lack of a method to combine the knowledge about the neurochemical mechanisms that occur in the molecular substrate of the brain with the whole brain haemodynamic response measured with fMRI has limited the investigation of the link between pharmacokinetic and pharmacodynamic processes. In this study we proposed a novel, multi-modal method to bridge this gap and explore the brain functional alterations induced by MDMA. The novelty of this approach relies on the fact that the density profiles of four serotonin receptors (i.e. 5-HT_1A_, 5-HT_1B_, 5-HT_2A_, and 5-HT_4_) and its transporter (5-HTT) were used to inform the rs-fMRI analysis in terms of the molecular distribution of serotonin in the brain in order to better characterise its functional response to MDMA, given its known serotonergic action. This method overcomes the limitations of the traditional data-driven and seed-based techniques used to explore rs-fMRI data in pharmacological challenge studies (Carhart-Harris et al., 2015; Roseman et al., 2014; Walpola et al., 2017). In fact, the ICA is generally used to extract common spatial patterns from heterogeneous data sets in order to investigate their intra- and inter-network connectivity, but the interpretation of the results can be challenging in the pharmacological context due to the complex structure of the functional networks and their interactions. By contrast, our approach defines a set of networks based on the *a priori* information about the distribution of specific targets to characterise the brain after a drug challenge, thus exploring the effects of pharmacological manipulation of brain networks from a molecular perspective. REACT shares with the seed-based approach the fact of being an *a priori* method. However, conventional seed-based analyses are typically limited to specific brain regions, the choice of which is qualitatively informed by the literature. Here instead, we guide our analysis with quantitative molecular data of voxel-wise distribution of the receptors across the brain. Moreover, with REACT we simultaneously include different targets in the model, taking into account the effects of all of them in the responses of interest.

In line with our hypothesis, we found that the networks showing significant changes after MDMA administration were the ones informed by the PET maps of 5-HT_1A_ and 5-HTT, which are the main targets of this compound. Specifically, the spatial maps enriched by the 5-HT_1A_ distribution information showed a decreased functional connectivity in several cortical areas, including the left and right precentral and postcentral gyri, the left frontal pole, insula, temporal and supramarginal gyri, inferior frontal gyrus, middle temporal gyrus, supramarginal gyrus, opercular cortex, Heschl's gyrus, and the right superior frontal gyrus, superior parietal lobule and supplementary motor cortex. The 5-HTT-related maps also showed connectivity changes localised in the anterior and posterior cingulate cortex, precuneus, intracalcarine and supracalcarine cortex, lingual gyrus and precentral gyrus. These findings partly overlap with those reported in other Arterial Spin Labelling (ASL) and fMRI studies of MDMA in healthy volunteers, i.e. an involvement of the visual cortex, supplementary motor areas, and posterior cingulate cortex (Carhart-Harris et al., 2015). Specifically, Carhart-Harris et al. (2015) suggested that the functional changes in these areas are related to a local stimulation of 5-HT_1B_ receptors, high localised in the subcalcarine region of the visual cortex (Varnas et al., 2011), due to an MDMA-induced release of serotonin. Here we were able to show that the MDMA effects on brain connectivity actually co-vary with the known distribution of the 5-HT_1A_ and 5-HTT targets. In addition, our target-enriched method was able to replicate previous findings but also extend MDMA effects on FC to other areas.

It is noteworthy that unlike the maps obtained with the traditional ICA analysis, the target-enriched FC maps are related to the plasma levels of MDMA: the higher the level of MDMA 45 min after drug administration, the higher the FC increase under MDMA in the right supramarginal gyrus, lateral occipital cortex and cuneal cortex. This suggests that these posterior cortical interactions with the areas of high 5-HT_1A_ directly capture the functional impact of drug exposure, which does not simply map onto the areas where the drug will bind directly.

Although the map enriched with the 5-HT_2A_ distribution did not show a main effect, it showed a significant correlation with the spiritual experience subscale. Very few studies have examined brain systems involved in spiritual experiences, but there is converging evidence for a role of the inferior parietal lobule, possibly related to an altered sense of self in relation to the environment (Miller et al., 2018). This is an area of particularly high density of 5-HT_2A_ receptors and the integration of activity of this and other areas of high 5-HT_2A_ density was with the left lingual gyrus, occipital and temporo-occipital fusiform gyrus, which are regions usually associated with the generation of mental imagery (Cooney et al., 2010). This result is remarkable as it highlights the role of the 5-HT_2A_ receptor, the principal target for hallucinogenic drugs, in one of the psychedelic components of the drug inducing subjective effects (Halberstadt, 2015). Moreover, it corroborates the hypothesis that the serotonin 2A receptor has a key role in the genesis of spiritual experiences (Carhart-Harris et al., 2014) and provides a methodology to link specific receptor targets to the neural mechanisms underlying subjective and behavioural outcomes.

The maps of connectivity enriched by each target may also have predictive value in other functions modulated by acute MDMA challenges such as response inhibition (Schmidt et al., 2017), where MDMA modulates activity in the right middle/inferior frontal gyrus and the superior parietal lobule. These areas overlap with the regions of connectivity enriched by the 5-HT_1A_ receptor and thus it is tempting to link the effects on response inhibition processing with 5-HT_1A_ receptor mediated effects, although a specific test of such a hypothesis will be required by including a wider battery of tests or through blockade of receptor subtypes.

We also explored whether or not overlapping expression of serotonergic targets had an effect on FC changes induced by MDMA using interaction terms in the general linear model. The results were largely similar to the main analysis for 5-HTT. However, there were some differences for the 5-HT_1A_-enriched maps. Taking into account the regional overlap of serotonin receptors, we detected an increased connectivity in the anterior cingulate gyrus, paracingulate gyrus, frontal pole, middle and superior frontal gyrus, all areas of particularly high density for the 5-HT_2A_ receptor relative to other subtypes. Notably, this analysis revealed changes for the 5-HT_1A_-5-HT_2A_ interaction but not for the 5-HT_1A_-5-HTT and 5-HT_2A_-5-HTT interactions. We note that the 5-HT_1A_ effects are generally inhibitory, stimulating the Gi/o cascade, whereas the 5-HT_2A_ stimulate the Gαq and Gβy cascades which are considered excitatory (Gonzalez-Maeso et al., 2007; Polter and Li, 2010). Despite these differential effects at the synaptic level, the interpretation of the interaction at the whole brain level is not straightforward. First, no thresholds were applied to the maps, so all regions were included even if the receptor density was very low. However, thresholded overlap maps require knowledge of where to set the thresholds for each receptor target. Second, the PET data only speak to regional co-localisation (i.e. voxel-wise) rather than cellular co-localisation. Third, the interaction effect may be more complex and involve other receptors, including non-serotonergic receptors. In fact, MDMA also increases extracellular levels of dopamine and noradrenaline, even if the clearest determinants of its effects are largely serotonergically-mediated. Nonetheless, the aim of the present study was to determine the utility of the PET receptor maps for the serotonergic system in guiding the connectivity analysis of MDMA effects. Future investigations should take into account the precise nature of interactions between the receptor targets, which are currently not known.

The results of the interaction analysis also showed a correlation of FC measures with oxytocin peripheral levels measured 165 min after the dose administration, confirming that there is a correlation between this neuropeptide and the functional response related to the 5-HT_1A_ receptor (Thompson et al., 2007). This correlation was localised in the brain stem, hippocampus and in the parahippocampal gyrus. A correlation between ΔOXT_165_ and MDMA-induced FC decrease was also found in the maps representing the interaction between 5-HT_1A_ – 5-HTT in the left frontal operculum cortex and among 5-HT_1A_ – 5-HT_2A_ – 5-HTT in the brainstem, right putamen and hippocampus. This is line with 5-HT and oxytocin together representing a functional interface for the regulation of emotion and behavior, with connectivity in brain stem and subcortical regions representing the functional effects of this interaction and supporting PET studies of interactions at the molecular level (Mottolese et al., 2014). The correlation with oxytocin at the sample time of 165 min is particularly relevant as it is in line with the timing of oxytocin release induced by MDMA, which has been shown to have a peak within the interval 60–240 min (Kirkpatrick et al., 2014). For the subjective effects, the correlation between the MDMA-induced FC decrease in the 5-HT_2A_-related maps and the spiritual experience subscale found in the main effects analysis was also found in the interaction analysis, potentially indicating the selectivity of this relationship.

With REACT, we provide a new method for exploring the changes in connectivity after accounting for the known binding profile of the drug. This novel approach has proven its effectiveness in combining the molecular knowledge from PET about the receptor distributions in the brain with the functional information from rs-fMRI data, providing a novel interpretation of the results in light of the MDMA affinity to serotonin. This is not the first multimodal study that integrates independent molecular atlases to functional data. The integration between haemodynamic markers and molecular imaging data is still a challenge. Some studies investigated the spatial association between the main effects of different drugs on the CBF and the molecular information from gene-expression, PET or autoradiography data using simple ROI-to-ROI correlations (Dukart et al., 2018; Selvaggi et al., 2018). While this approach has proven its efficacy with ASL data sets, it would be over simplistic if applied to rs-fMRI data, since the temporal dynamics of the BOLD signal would be overlooked. Deco et al. (2018) tackled this problem by applying an excitation/inhibition (E/I) neuro-modulatory model combined with the 5-HT_2A_ receptor density map from (Beliveau et al., 2017), fMRI and diffusion data and applied this to model the functional changes with lysergic acid diethylamide (LSD) in healthy participants. Similarly, we addressed this issue by combining rs-fMRI with the underlying neuroreceptor distributions in order to clearly link the functional response to a specific drug (i.e., the MDMA) with the molecular substrates. However, the main difference in REACT compared to the approach developed by Deco and colleagues is the weighting of the resting state signal on the basis of the molecular distribution of multiple serotonin receptor targets across the brain, without taking into account any *a priori* neuro-modulatory model of the E/I balance.

Further specification from intra-regional variation across subjects is not possible using the current dataset as it requires PET data for each ligand from each participant, but it would be interesting to test whether the high-resolution template is fully capable of estimating the subject-specific functional response, or if the subject-specific receptor density maps would provide additional information. This may be of particular importance in long-term MDMA users who may have an altered distribution of receptor densities, with a recent meta-analysis supporting widespread reductions in 5-HTT density (Muller et al., 2019).

Ongoing work is also validating this approach with other compounds (i.e. antipsychotics, psilocybin). However, high-resolution templates representing the density profiles of other receptors such as dopamine will be needed in order to standardise the analysis and test other compounds with different affinities.

## 5 Conclusion

Supported by the receptor occupancy theory, which states that the magnitude of the drug response is a function of receptor availability and drug binding, the multimodal approach proposed in this study has proven that there is a link between the haemodynamic response to MDMA and the underlying neuroreceptor distribution and showed that it is possible to utilise receptor maps to enrich connectivity analyses of drug effects. Assuming that drugs modulate the neuronal activity in brain regions that have a preferred neurotransmitter affinity (e.g. higher receptor density) for them, this new approach defines the drug-specific topography of brain functional connectivity and may provide an interesting new fingerprint in the characterisation of novel compounds and potentially greater insight to the commonly observed eclectic response to treatment.
