# Supplementary material for: Receptor-Enriched Analysis of functional connectivity by targets (REACT): A novel, multimodal analytical approach informed by PET to study the pharmacodynamic response of the brain under MDMA
Source: Neuroimage. 2019 Jul 15;195:252–60. doi: 10.1016/j.neuroimage.2019.04.007 (PMC6547164; doi:10.1016/j.neuroimage.2019.04.007)
Supplement: Multimedia component 1 [file mmc1.docx]

**Receptor-Enriched Analysis of functional Connectivity by Targets (REACT):**

**A novel, multimodal analytical approach informed by PET to study the pharmacodynamic response of the brain under MDMA**

Dipasquale et al.

**SUPPLEMENTARY MATERIAL**

**RESULTS**

**Interaction analysis**

By including in the two-step multivariate analysis only the targets with a known affinity for MDMA (i.e. the 5-HT_1A_, 5-HT_2A_, 5-HTT) and taking also account of their interactions (5-HT_1A_ – 5-HT_2A_, 5-HT_1A_ – 5-HTT, 5-HT_2A_ – 5-HTT and 5-HT_1A_ – 5-HT_2A_ – 5-HTT), consistent results with the five-target analysis were found for the 5-HT_1A_ receptor and the 5-HTT transporter, namely a significant FC decrease induced by MDMA (p_FWE_ < 0.05, corrected for multiple comparisons at the cluster level using the threshold-free cluster enhancement (TFCE) option, Bonferroni corrected for multiple comparisons across maps and contrasts). For the 5-HT_1A_-derived maps, the FC reduction involved the temporal gyrus, angular gyrus, lateral occipital cortex, precuneous, lingual gyrus, temporal occipital fusiform cortex, left middle frontal gyrus, left precentral and postcentral gyrus, left superior parietal lobule, left supramarginal gyrus, left central opercular cortex, left and right hippocampus, right accumbens, right supramarginal gyrus, right intracalcarine and supracalcarine cortex, right cingulate gyrus, right cuneal cortex, right occipital pole. The decrease in the 5-HTT-derived maps was mainly localised in the precuneous, cuneal cortex, lateral occipital cortex and supracalcarine cortex.

The interaction analysis also highlighted a significant FC increase after MDMA (p_FWE_ < 0.05, TFCE corrected for multiple comparisons at the cluster level, Bonferroni corrected for multiple comparisons across maps and contrasts) in the 5-HT_1A_-enriched maps in the paracingulate gyrus, frontal pole, anterior cingulate gyrus, middle and superior frontal gyrus, and a FC decrease in the maps derived by the HT_1A_ – 5-HT_2A_ interaction, specifically involving the Heschl's gyrus, opercular cortex, left frontal pole, left insular cortex, left inferior frontal gyrus, left precentral and postcentral gyrus, left frontal orbital cortex, right superior and middle temporal gyrus, right supramarginal gyrus, right angular gyrus, right lateral occipital cortex, right lingual gyrus. All the results are showed in the Supplementary figure 1.


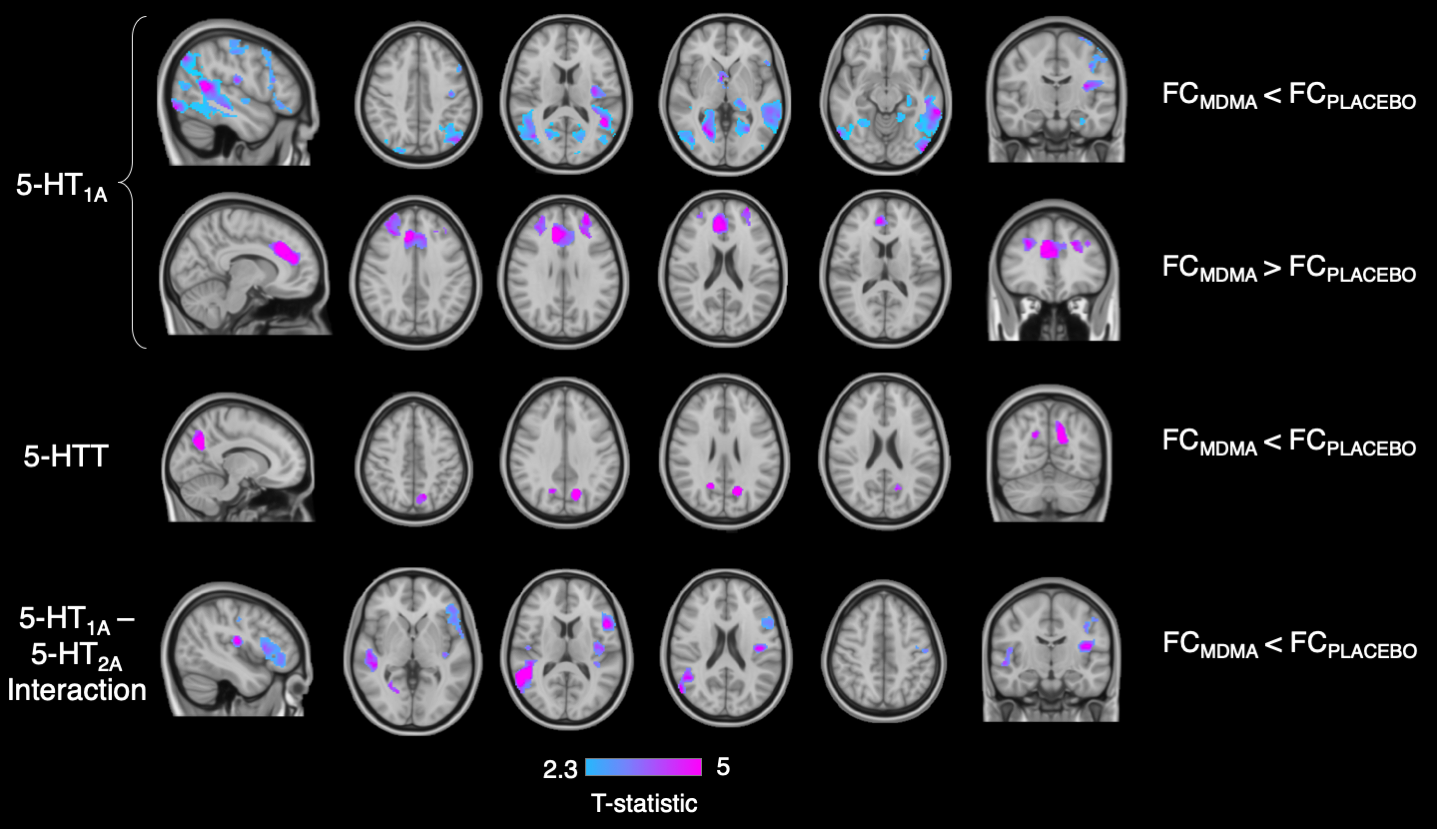


Supplementary Figure 1. Functional connectivity (FC) changes after MDMA in the PET-enriched maps. An MDMA-induced functional connectivity decrease involves the 5-HT_1A_, 5-HTT-enriched maps and the ones resulting from the interaction between the 5-HT_1A_ and 5-HT_2A_ maps. A connectivity increase after MDMA is also showed in the 5-HT_1A_-enriched maps.

**Resting State Network analysis**

Thirteen components out of the twenty estimated with the ICA were labelled as RSNs and classified as the primary, medial and lateral visual networks, default mode network, sensorimotor network, auditory network, salience network, task positive network, ventral stream, right lateral network, medial temporal network, thalamus and cerebellum (Supplementary figure 2).


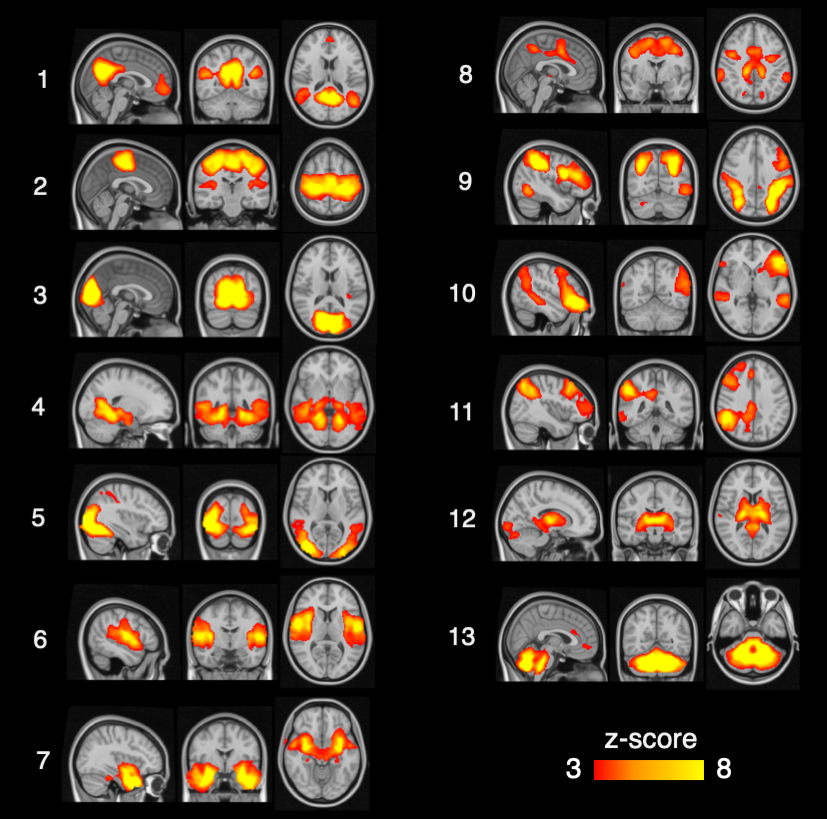


Supplementary Figure 2. Resting State Networks (RSNs) estimated with the ICA on an independent dataset. 1. Default mode network; 2. sensorimotor network; 3. primary visual network; 4. medial visual network; 5. lateral visual network; 6. auditory network; 7. medial temporal network; 8. salience network; 9. task positive network; 10. ventral stream network; 11. right lateral network; 12. thalamus; 13. cerebellum.

A significant FC decrease after MDMA compared to placebo was found in the primary visual and sensorimotor networks, while the medial temporal, salience and ventral stream networks reported an increased FC (p_FWE_ < 0.05, TFCE corrected for multiple comparisons at the cluster level). However, nothing survives Bonferroni correction for multiple comparisons across all the RSNs and contrasts.

**Correlation with behavioural measures, MDMA and oxytocin plasma levels**

A FC decrease after MDMA in the 5-HT_2A_-enriched maps correlated with the spiritual experience subscale (r = -0.831, p < 0.0001, 95% CI = -0.926 to -0.642; Supplementary figure 3A). As regards the correlation with the pharmacokinetic measures, no correlations were found with the MDMA plasma levels, while Significant correlations with increased oxytocin plasma levels after MDMA administration were found with MDMA-induced FC decreases in some regions of the 5-HT_1A_ (r = -0.820, p < 0.0001, 95% CI = -0.930 to -0.570), HT_1A_ – 5-HTT (r = -0.868, p < 0.0001, 95% CI = -0.962 to -0.662) and HT_1A_ – 5-HT_2A_ – 5-HTT maps (r = -0.881, p < 0.0001, 95% CI = -0.950 and -0.657) (Supplementary figure 3B).

As to the RSNs, no significant correlations were found with the MDMA and oxytocin peripheral levels and with the subjective subscales.

Supplementary Figure 3. Correlations between behavioural measures/oxytocin plasma levels and functional connectivity for the maps estimated in the interaction analysis. Panel A: the FC decrease after MDMA in the 5-HT_2A_-enriched maps shows a significant correlation with spiritual experience subscale. Panel B: the oxytocin peripheral levels collected 165 minutes after MDMA administration are significantly correlated with the MDMA-induced FC decrease in the 5-HT_1A_ -enriched maps and in those resulting from the interaction between 5-HT_1A_ and 5-HTT and among 5-HT_1A_, 5-HT_1A_ and 5-HTT.
